# The etiological relationship between the general factors of psychopathology and personality; a longitudinal twin study from adolescence into young adulthood

**DOI:** 10.3389/fpsyg.2025.1564305

**Published:** 2025-07-08

**Authors:** Trine Waaktaar, Eirunn Skaug, Svenn Torgersen

**Affiliations:** Department of Psychology, University of Oslo, Oslo, Norway

**Keywords:** psychopathology, personality, general factors, adolescent development, twin study

## Abstract

**Background:**

Mental disorders and normal personality are interconnected domains. Recent studies highlight the dimensional and hierarchical nature of psychopathology and personality, focusing on their general factor levels. However, their relationship remains unclear.

**Aims:**

This study explored the etiological relationship between the general factors of psychopathology and personality from adolescence to young adulthood.

**Methods:**

Longitudinal data from seven national twin cohorts (*N* = 1,538 pairs) were collected across three waves (ages 12–22). Data was analyzed using a genetically informative random intercept cross-lagged panel model and Cholesky decomposition modeling.

**Results:**

Negligible cross-lagged effects were observed between the general factors. Both showed substantial stability, with genetic influences explaining most of the time-invariant variance. About one-quarter of genetic stability was shared.

**Conclusion:**

Psychopathology and personality are distinct yet parallel domains developing through adolescence into young adulthood. Notably, change in one did not lead to change in the other.

## Introduction

Psychopathology and personality each capture essential aspects of human adaptation. While psychopathology focuses on the manifestation and study of mental disorders and maladaptive behaviors, personality encompasses the enduring traits and patterns of thoughts, emotions, and behaviors that define an individual. The two domains are closely interrelated. Positive correlations have consistently been reported between personality traits such as neuroticism and conditions of depression (Widiger and Trull, 1992), anxiety and substance use disorders (Kotov et al., 2010; Ruiz et al., 2008). Similarly, low scores on extraversion (Jylhä et al., 2009), conscientiousness and (to a lesser extent) agreeableness have been systematically linked to various mental disorders such as ADHD, substance use, and various internalizing disorders (Malouff et al., 2005; Trull and Sher, 1994). Openness has been particularly, albeit not consistently, related to thought disorders and psychotic illness (Boyette et al., 2013; Ristić et al., 2023).

Several models depicting the nature of the psychopathology—personality relationship have been proposed (Tackett, 2006; Widiger, 2011). Although some item overlap exists on facet and criterion level, this does not suffice to explain the extent of the observed association between the two concepts (Uliaszek et al., 2009; Williams et al., 2010). Focusing on associations, some have hypothesized that psychopathology symptoms and personality traits belong to the same continuum. In this view, psychopathology could represent the extreme end of normal personality traits (Nicolson et al., 2003). Alternatively, personality might more broadly serve as a common factor explaining shared variance across several mental illness symptoms or diagnoses. An example would be if neuroticism were considered a common factor in all internalizing problems (Griffith et al., 2010). While such models emphasize associations and conceptual overlap, etiological hypotheses regarding the underlying causal structure behind the dual psychopathology—personality relationship, have also been proposed.

Psychopathology and personality may be etiologically linked in at least two ways. They may share a common causal basis, such as being influenced by the same genetic or environmental factors. This could be the case with the vulnerability model, which suggests that certain personality traits (e.g., neuroticism) predispose individuals to developing mental illnesses (e.g., depression) (Xia et al., 2011). Such a risk factor model does not necessarily imply causality (Hengartner et al., 2016; Lynch et al., 2021; Ormel et al., 2013). More direct causal models imply that psychopathology and personality exist as separate unities that unilaterally or mutually influence each other's development (Ormel et al., 2013; Widiger, 2011). Traditional pathoplasticity, scar, and complication models all involve hypotheses about causality. Within a pathoplasticity model, personality traits can affect the course, severity, and treatment outcomes of psychopathological conditions. Experiencing psychopathology can potentially also leave lasting changes (thus often called a “scar” model), or otherwise disrupt or complicate the natural progression of an individual's personality development.

The above mentioned etiological models are not mutually exclusive (Kendler et al., 1993), and several causal pathways may be operating in parallel. Identifying and understanding the nature of the relationship is crucial for increasing our understanding of the individual's functioning, as well as from a practical viewpoint. Early assessment and targeting of one dimension could potentially be utilized to prevent or influence the development of problems in the other (Etkin et al., 2022). However, several methodological limitations may have contributed to slow progress in the study of the nature of the psychopathology—personality relationship (Tackett, 2006). Most research to date has been based on cross-sectional studies of adult samples. Longitudinal studies and developmental perspectives that could capture the temporal predictive and developmental relationships between the two domains are highly called for (Durbin and Hicks, 2014; Wilson and Olino, 2021).

One notable limitation of current studies of the nature of the psychopathology—personality relationship has been its reliance on traditional clinical diagnostic systems. Methodological and conceptual advances in the field of psychopathology the last decennium strongly advocate for a shift from discrete descriptive diagnostic entities based on clinical consensus to empirically founded dimensional measures (Haslam et al., 2020; Lahey et al., 2004; Plomin et al., 2009). Adopting a dimensional perspective on psychopathology offers significant research advantages. It allows the use of established quantitative methods to study the widespread comorbidity (Kessler et al., 2005), correlations observed over time (Caspi et al., 2020) and associations across generational lines (Zhou et al., 2023) in common mental disorders.

Using a factor analytic approach on mental health data, Lahey et al. (2012) reported support for a general factor accounting for the shared variance in 11 prevalent mental disorders in a national representative adult sample. Factor analysis had already been successfully applied to explore the inherent hierarchical structure of dimensionally measured complex traits like intelligence (Spearman, 1904) and personality (Costa and Mccrae, 1992). From the study of intelligence, the existence and utility of a general intelligence factor, G, explaining all common variance among cognitive abilities has been discussed and has demonstrated its utility (Spearman, 1904). Personality research (John et al., 1988) has long debated the optimal number of factors necessary to account for human variability in fundamental adaptive characteristics. Although the Big Five model (Mccrae and Costa, 2008) has achieved considerable recognition, the Big One or the General Factor of Personality (Erdle and Rushton, 2011; Musek, 2007; Rushton et al., 2009) is among several hierarchical models that has also received empirical support.

Caspi et al. (2014), Caspi and Moffitt (2018), and others (Wright et al., 2013) strongly argued for an exploration of a general factor in psychopathology. Accumulating evidence on the dimensionality, comorbidity, and predictive relationships of mental disorders suggests that a general factor approach may improve prediction of functional impairment compared to narrower perspectives. The general psychopathology factor *(p)* has since been the object of considerable interest and research activity, and it bears several indications of a meaningful concept. Studies using different measurement and statistical approaches, have found that the *p* factor exhibits superior model fit across samples (Scopel Hoffmann et al., 2022) and structural stability (measurement invariance). This makes it a relevant framework for measuring transdiagnostic change over time (Gluschkoff et al., 2019) and across developmental phases (Mcelroy et al., 2018; Murray et al., 2016; Snyder et al., 2017). The Hierarchical Taxonomy of Psychopathology (HiTOP) initiative is one of the most advanced and comprehensive systems developed to date. It substantiates its proposed hierarchical structure with evidence from multiple research traditions, such as neurobiology, genetics, and both clinical and normal personality research. They propose a general psychopathology factor at the top of a comprehensive hierarchical model of mental illness, which includes levels from individual symptoms and syndromes to subfactors, spectra, and superspectra (Kotov et al., 2021).

As expected, the general factor initiative within psychopathology as well as within personality research has generated several discussions about measurement, analysis and interpretation of the approach (Davies et al., 2015; Harris et al., 2024; Littlefield et al., 2021). There are many ways to the technical and statistical challenges of specifying and interpreting implications of different measurement models (Fried et al., 2021; Watts et al., 2019), for a comprehensive review see Markon (2019). Caspi et al. (2024) in a recent review of the last 10 years development within the field, argue against maintaining a too narrow focus on the measurement model and statistical aspects, and focus on criterion-validating research designs.

Notwithstanding the challenges in general factor approaches, there is a growing acknowledgment that the traditional narrow, trait-by-trait and illness-by-illness research and intervention strategies may be contributing to limiting progress in our understanding of the mutual relationship between the two domains (Caspi et al., 2014, 2024; Krueger and Tackett, 2003). As noted by Rosenström et al. (2019), using general factor models for psychopathology and personality offers scientific simplicity and clinical value by treating psychiatric comorbidity as a quantifiable construct rather than an unexplained association.

Consequently, more comprehensive knowledge about the nature of the relationship between psychopathology and personality on a general level is needed. Despite theoretical and conceptual as well as cross sectional empirical evidence for associations between psychopathology and personality in adult samples, there is a need for more developmentally oriented and genetically informative designs that can capture the etiological structure behind their relationship (Briley et al., 2018; Durbin and Hicks, 2014; Wilson and Olino, 2021). The transition from youth to young adulthood is especially interesting for longitudinal studies on developmental change. A significant proportion of mental disorders manifest during adolescence (Cicchetti and Rogosch, 2002; Dalsgaard et al., 2020; Kim-Cohen et al., 2003; Ormel et al., 2015; Paus et al., 2008). It is also a period when personality development is still in relative flux (Roberts et al., 2006), reaching its typically high stability in young adulthood (Bleidorn et al., 2022; Lüdtke et al., 2009; Schwaba and Bleidorn, 2018). Thus, adolescence offers a unique window for empirically examining change in these otherwise relatively stable traits.

The present longitudinal study measured general psychopathology and personality in a large population of adolescent twins in three waves from early adolescence to young adulthood. The overreaching aim was to examine the etiological relationship between psychopathology and personality. Specifically, we aimed to address two key research questions. First, to what extent does change in personality predict change in psychopathology, and vice versa? Second, how stable are psychopathology and personality over time, and to what extent is this stability influenced by common genetic and environmental factors? Through these investigations, we aim to contribute to the understanding of the relationship between psychopathology and personality, providing insights into both their mutual influence and inherent stability.

## Materials and methods

### Ethics, transparency, and openness

This longitudinal study started in 2005, thus, before any preregistration for epidemiological data was organized or expected. Data collection received preapproval in 2005 from the Norwegian Data Protection Authority, mandating a 20-year period of individual data protection followed by either data deletion or anonymization. Anonymized data will be available upon request after 2025.

Open Mx R code for Cholesky decomposition modeling is publicly available several places, e.g., Neale and Maes (2004) and http://hermine-maes.squarespace.com/.

We report how we determined our sample size, all data exclusions, all manipulations, and all measures in the study. Descriptive statistics, twin correlations and fit statistics from the trivariate Cholesky decompositions are provided in Supplementary material posted on the journal's Web site.

The study was approved by the Norwegian Data Inspectorate and the Regional Committees for Medical and Health Research Ethics, Ref. 2015/4 (19661). The research adhered to the ethical principles outlined by the American Psychological Association and the Declaration of Helsinki, 2004 version.

### Study design

Data was collected as part of the Oslo University Adolescent and Young Adult Twin Project (Torgersen and Waaktaar, 2019, 2020). This study employed a repeated measures twin design, which enables the investigation of stability and change in the measured phenotypes over time, as well as determining the role of genes and the environment in such processes. Since monozygotic (MZ) twins share 100% of their genes, and dizygotic (DZ) twins share, on average, 50% of their segregating genes—one can effectively differentiate between genetic and environmental influences.

### Sample and procedure

The Norwegian Medical Birth Register provided information on all multiples born in Norway between 1988 and 1994, totaling 5,374 multiple births (10,748 individual twins). A postal invitation was sent to the 4,669 twin pairs who were alive and living in the country (elective pairs) at the study's start. Informed consent was given by 2,486 pairs. Data for the present study were collected via self-report questionnaires sent to the twins at three different times during adolescence, beginning when the participants were aged 12–18 years. A total of 1,393 pairs (29.8% of elective pairs) participated in the first wave, 1,065 (22.8%) in the second wave, and 883 (18.9%) in the third. According to questionnaire response dates, the median time span between waves was 1.8 years between Waves 1 and 2 (94.4% between 1.5 and 2.5 years), 2.6 years between Waves 2 and 3 (99.6% between 2.0 and 3.0 years), and 4.4 years between Waves 1 and 3 (96.7% between 4.0 and 5.0 years). For further details on recruitment, participation, demographic characteristics, and dropout rates, see (Torgersen and Waaktaar, 2019; Waaktaar and Torgersen, 2012).

Thirty-three percent (*n* = 1,538 pairs) of the elective pairs had at least one twin respond to at least one measure of general psychopathology and general personality on at least one occasion. Of these pairs, 577 were monozygotic and 961 were dizygotic, both same and opposite sex. In 697 pairs, both twins provided valid answers on all three substance measures across all waves (complete pairs). Longitudinal attrition analyses showed no significant differences in zygosity and sex distribution between complete pairs and any pairs (complete plus incomplete) for all waves.

### Zygosity determination

Zygosity was determined using a combination of questionnaire data on twin physical similarity and DNA samples obtained through cheek swabs from a subset of participants. This procedure resulted in a classification accuracy rate of >99% (for details, see Skaug et al., 2022a; Torgersen, 1979; Torgersen and Waaktaar, 2019; Waaktaar and Torgersen, 2012).

### Measures

The general factors of psychopathology and personality used in this study were derived from factor analyses (see the Analyses Section below) of established dimensional measures of mental disorder symptoms and the five-factor model of personality traits (Torgersen and Waaktaar, 2019, 2020). All scales used in the survey were abbreviated versions for increased response rates. The items chosen for each scale were selected through a two-phase pilot testing process on two independent school-based adolescent samples, with the items demonstrating the highest item-to-scale correlations being chosen due to their superior psychometric properties. The measures entered into the factor analyses are briefly presented below, see more details and the specifics of their abbreviated versions in earlier publications from the Oslo University Adolescent and Young Adult Twin Project, see (Ask et al., 2014, 2016; Kandler et al., 2019; Seglem et al., 2015; Skaug et al., 2024, 2022b; Torgersen and Waaktaar, 2019; Waszczuk et al., 2019).

#### Psychopathology

The general psychopathology factor was constructed on the basis of self-report of symptoms of 7 mental disorders prevalent in adolescence. These were depression, anxiety, somatoform, eating difficulties, delinquency, conduct disorder and substance abuse.

##### Depressive symptoms

Depressive symptoms were measured by the average score of 8 items selected from the originally 20 items Center of Epidemiological Studies Depression Scale (Radloff, 1977). The respondents were asked how often they experienced symptoms during the last 12 months on a 4-point scale ranging from 0 (*almost never*) to 3 (*most of the time*).

##### Anxiety symptoms

Anxiety symptoms were measured by the average score of 10 items from the Screen for Child Anxiety-Related Emotional Disorder questionnaire (SCARED) (Birmaher et al., 1997). The questionnaire originally comprised 38 items based on the DSM-IV anxiety subtypes Generalized Anxiety Disorder, Panic disorder, Social Anxiety disorder and Separation Anxiety disorder. Symptom severity over the past 12 months was rated on a 3-point scale: 0 (*not true or hardly ever true*), 1 (*sometimes true*), and 2 (*true or often true*).

##### Somatic complaints

Somatic complaints were assessed using a subset of 10 items from the 28-item Children's Somatization Inventory (Garber et al., 1991). Subjects were asked to rate the extent of discomfort caused by various symptoms—such as headaches, faintness or dizziness, heart or chest pains, muscle soreness, hot or cold spells, localized body weakness, nausea or upset stomach, stomach pain, joint weakness, and joint pain—experienced by the twins in the past 12 months. Ratings were given on a 5-point scale, where 0 indicated “*not at all*” and 4 indicated “*very much*.”

##### Eating disorder symptoms

Eating disorder symptoms were assessed at each time point using 11 items from the Eating Disorder Inventory-Revised (EDI-R) (Garner, 2004). The selected items included three from the Drive for Thinness subscale, four from the Bulimia subscale, and four from the Body Dissatisfaction subscale. Participants reported how often they experienced each symptom over the preceding 12 months on a scale from 0 (*never*) to 5 (*always*). The responses were summed and averaged to create a total Eating disorder score.

##### Delinquency

Participants were asked to report the frequency of committing nine different forms of law- or rule-breaking behaviors over the past 12 months (Leblanc and Tremblay, 1988). The scale included items addressing physical fights, stealing, carrying weapons, vandalism, and other disobedient behaviors such as staying out late when supposed to be home. Responses were recorded on a 4-point Likert scale ranging from 0 (*never*) to 3 (*very often*). Average scores were then computed to derive a total delinquency score.

##### Conduct problems

Conduct problems over the past year were assessed using the Conduct Problems Scale, a subscale of the Strengths and Difficulties Questionnaire (SDQ) (Goodman, 1997, 2001). This 5-item scale includes questions about temper, fighting, lying, cheating, and stealing. Participants responded on a 3-point Likert scale ranging from 0 (*not true*) to 2 (*certainly true*). Average scores were calculated, with higher scores indicating a greater number of conduct problems.

##### Substance abuse

Substance abuse was measured with the six-item CRAFFT screening test (Knight et al., 2002, 1999). Items were on the format of “Have you ever ridden in a car driven by someone (including yourself) who was ‘high' or had been using alcohol or drugs?”, qualified with “the past 12 months,” and answered in a yes/no format. A total scale score ranging from 0 to 6 was obtained by summing the number of yes-responses.

#### Personality

The general personality factor was generated on the basis of an abbreviated 40-item version of the Hierarchical Personality Inventory for Children (HiPIC) (Mervielde and De Fruyt, 1999). The HiPIC is a widely utilized tool for assessing the Big Five traits in children and adolescents, and includes emotional stability (reverse of neuroticism), extraversion, imagination (analogous to openness), benevolence (analogous to agreeableness), and conscientiousness. Participants rated items on a 5-point Likert scale ranging from 0 (*not typical*) to 4 (*very typical*). Each personality trait was quantified by calculating the average score of the eight items corresponding to each of the five traits.

### Statistical analyses

Missing data were imputed using multiple imputation by fully conditional specification (Van Buuren, 2007). The mean scores based on 10 iterations were used as input variables in the factor analyses.

First, we performed a factor analysis with one factor on the Big five personality traits. Specifically, three general personality factors were created, each based on the measures of the Big Five traits at each of the three measurement waves. The factor loadings for the general personality factors were constrained to be equal across the measurement waves. Maintaining constant factor loadings ensures conceptual consistency by assuming that the structure of personality traits does not change over time. This allows us to attribute observed changes in the general personality factor to real changes in personality rather than varying influences of individual traits. Additionally, this approach reduces model complexity and helps control for potential measurement error that might vary across time points. By setting the factor loadings to be equal, we ensure that our longitudinal analyses capture actual stability and change in the general personality factor, thereby enhancing the validity and interpretability of our results. A similar approach was employed for the psychopathology measures. Specifically, three general psychopathology factors were created, each based on the measures of psychopathology at each of the three measurement waves. These factor analyses served as initial steps to generate factor scores. Specifically, the factor scores of the general personality factors and the general psychopathology factors (i.e., one factor score for personality and one for psychopathology at each wave) were used in subsequent analyses.

Next, phenotypic correlations were computed to examine the stability of psychopathology and personality over time, as well as the association between them. Cross-twin correlations were then calculated to provide initial impressions of the genetic and environmental contributions to variation within, and the covariance between, psychopathology and personality.

Twin studies make use of the fact that MZ twins are genetically identical, while DZ twins share, on average, half of their segregating genes. This genetic difference allows us to decompose the variance of an observed phenotype (and the covariance between phenotypes) into three sources. Additive genetic influences (A; the effect of genes that operate in an additive manner) are inferred by the extent to which the correlation between MZ twins is higher than the correlation between DZ twins. Shared environmental influences (C; environmental factors contributing to phenotypic similarity among family members) are inferred when the correlation between DZ twins exceeds half of the MZ correlation. Any remaining variance or covariance, not explained by A and/or C, is attributed to non-shared environmental influences (E; any factors contributing to phenotypic differences among family members, including measurement error).

The correlation analyses were extended using a series of multivariate twin models. The structural equation modeling R package OpenMx, was used for the multivariate twin models (Neale et al., 2016). First, to partition the observed phenotypic variances in the psychopathology and personality factors into genetic (heritability) and environmental components, we fitted two trivariate Cholesky decomposition models to data from the three measurement waves, separately for psychopathology and personality. In a Cholesky decomposition, one set of latent genetic and environmental factors (A, C, and E) is specified for each variable, with the first set loading on all variables, the second set loading on all variables except the first, and so on. The Cholesky decomposition is among the most widely used twin models, offering a robust approach to estimate genetic and environmental sources of variance and covariance with minimal theoretical assumptions (Neale and Maes, 2004). For both psychopathology and personality, we first fitted a full ACE model, followed by a reduced AE model. To account for sex differences in mean level, separate means were estimated for males and females. Model fit was determined by comparing the models' Akaike's information criterion (AIC; Akaike, 1987) and Bayesian information criterion (BIC; Raftery, 1995), with lower values indicating better model fit.

**Figure 1:**
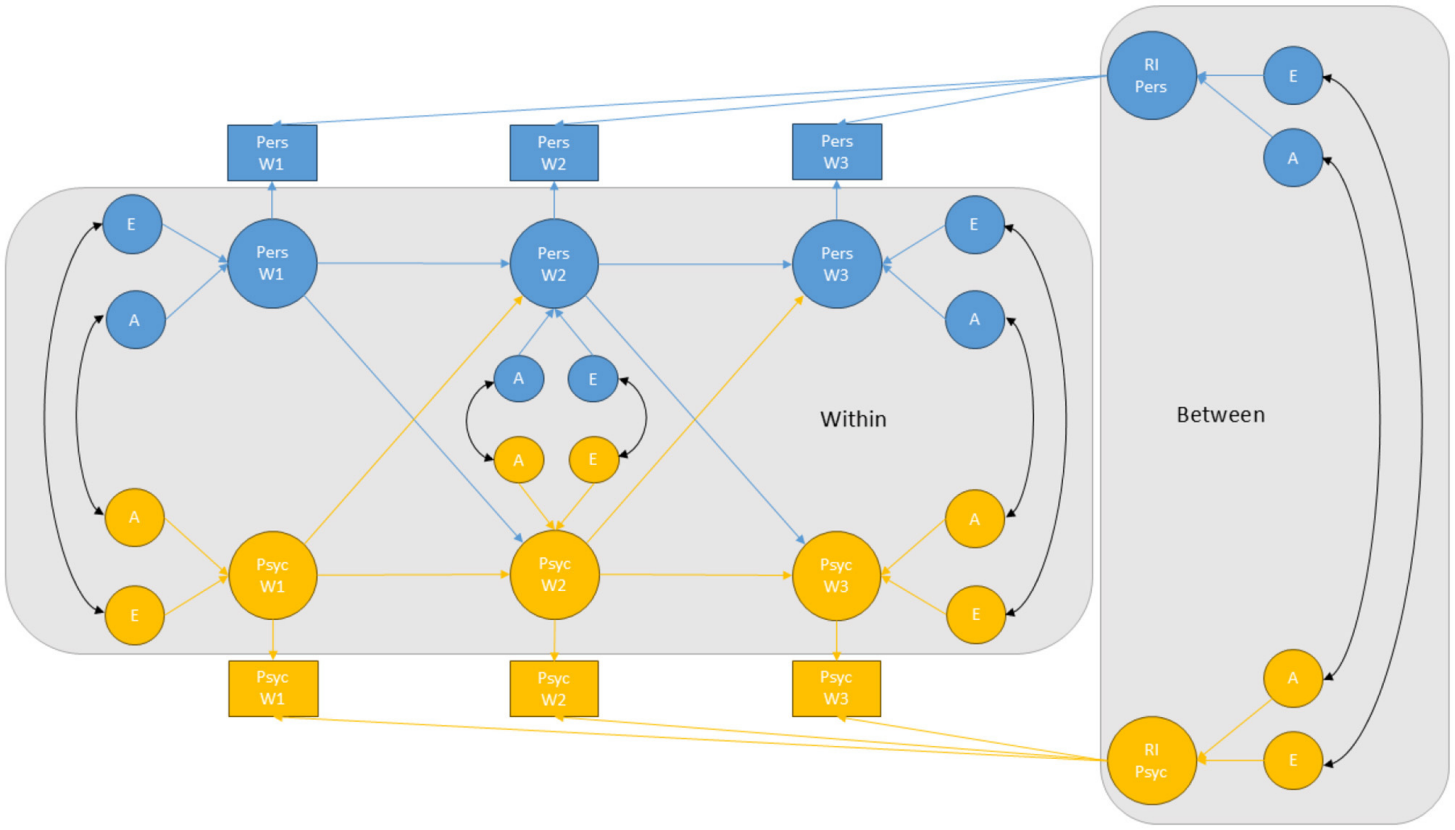
Illustration of the genetically informative random intercept cross-lagged panel model for the relationship between psychopathology and personality. Pers, personality; Psyc, psychopathology; RI, random intercept; A, additive genetic factors; E, non-shared environmental factors; W1, W2, and W3, measurement wave 1, 2, and 3, respectively. For simplicity, the model is shown for one twin only, and only A and E influences are included. Rectangles represent observed variables and circles represent latent variables. “Between” indicates the between-person processes (time-invariant stability). “Within” indicates the within-person processes (variance due to changes within individuals over time), which are composed of autoregressive paths (temporal stability) and cross-lagged paths (change). Factor loadings from the random intercepts to the observed variables were constrained to one to reflect time-invariant effects. Within-person latent factors for psychopathology and personality were modeled by specifying a latent variable for each observed variable, with all factor loadings constrained to one.

Second, a genetically informative random intercept cross-lagged panel model (RI-CLPM) was fitted to data. The RI-CLPM was modeled following procedures as described by Hamaker et al. (2015). Additionally, we extended the RI-CLPM by partitioning the variances into genetic and environmental sources of variance, and by modeling genetic and environmental correlations. The modeling procedure is explained in detail in Skaug et al. (2024). The effects of sex were controlled for by regressing out the effects of sex from each measure (i.e., psychopathology and personality). That is, the residuals from models where psychopathology and personality were predicted from sex were used as input variables in the cross-lagged models. Absolute model fit was assessed by examining the Comparative Fit Index (CFI), the Tucker-Lewis Index (TLI) and the Root Mean Square Error of Approximation (RMSEA). CFI and TLI values >0.95 and RMSEA values <0.06 were considered as indicating good model fit (Hu and Bentler, 1999). A figurative illustration of the model is provided in Figure 1.

To address our first research question, we examined the within-person processes. Specifically, we investigated whether within-person changes in personality predicted within-person changes in psychopathology, and vice versa (i.e., the cross-lagged paths). For example, a significant cross-lagged path from personality to psychopathology implies that within-person changes in personality (i.e., individuals' deviations from their own stable level/score on personality) predict within-person changes in psychopathology. The significance of the cross-lagged paths was tested by sequentially fixing each cross-lagged parameter to zero. These reduced models were then compared to the full RI-CLPM by likelihood ratio chi square (χ^2^) tests. A non-significant χ^2^ difference suggests that the restricted model does not result in a significant loss of fit.

Next, to address our second research question, we examined the time-invariant stability (i.e., the random intercepts) in psychopathology and personality. Specifically, we examined the level of stability in these constructs, the influence of genetic and environmental factors on the stability, and the nature of the association between stable traits of psychopathology and personality. These investigations provide insights into how genes and the environment contribute to within-trait stability and to the interplay between stable traits of psychopathology and personality.

To further explore the etiological relationship between stable traits of psychopathology and personality (i.e., our second research question), we fitted bivariate Cholesky decomposition models. While the RI-CLPM inform us about genetic and environmental correlations between the random intercepts (i.e., stable variance in the constructs), it does not estimate the proportion of common genetic and environmental variance underlying the stability of the constructs. To address this, we created aggregated measures of psychopathology and personality across the measurement waves and fitted bivariate Cholesky decompositions models to these composite scores. To create an aggregated psychopathology measure, we conducted a factor analysis, extracting one factor based on the psychopathology measures at the three different time points (i.e., the variables shown in rectangles in Figure 1). This analysis allowed us to combine multiple time-point measurements into one factor score. By extracting a single factor, we captured the shared variance across the measurement waves, resulting in a composite score that reflects the stability in psychopathology. A similar approach was applied to personality. Subsequently, we estimated the extent of common genetic and environmental variance underlying stable traits of psychopathology and personality by fitting bivariate Cholesky decomposition models to these aggregated factor scores.

## Results

Descriptive statistics for all study variables are presented in Supplementary Table S1.

In our initial analyses, factor analyses were performed to create a general factor of personality and a general factor of psychopathology at each measurement wave. The factor loadings are provided in Table 1. As described in the Method Section, the factor loadings were set to be equal across measurement waves, resulting in five consistent loadings across the three time points. All of the Big Five traits, except Agreeableness, showed high loadings on the general personality factor, this factor explaining 44% of the total variance in the five personality traits. The one-factor solution of the seven psychopathology traits showed factor loadings ranging from 0.40 to 0.80, with the general psychopathology factor explaining 43% of the variance in these traits.

**Table 1:** Factor loadings.

**Factor**	**Factor loading**
**General factor of personality**	
Neuroticism	−0.55
Extraversion	0.71
Openness	0.65
Agreeableness	0.25
Conscientiousness	0.53
**General factor of psychopathology**	
Depressive symptoms	0.80
Anxiety symptoms	0.67
Somatic complaints	0.73
Eating disorder symptoms	0.58
Delinquency	0.41
Conduct problems	0.41
Substance abuse	0.40

Phenotypic correlations between the psychopathology and personality factors are given in Table 2. High correlations were observed within each factor across waves, with correlations typically decreasing with increased time lag between measurement points. This pattern would indicate notable stability in both psychopathology and personality over time. The moderate negative correlations found between the factors across all time points implied a significant and inverse relationship between psychopathology and personality.

**Table 2:** Phenotypic correlations.

	**1. Personality _Wave 1_**	**2. Personality _Wave 2_**	**3. Personality _Wave 3_**	**4. Psychopathology _Wave 1_**	**5. Psychopathology _Wave 2_**
1. Personality_Wave 1_	–				
2. Personality_Wave 2_	0.69^***^	–			
3. Personality_Wave 3_	0.64^***^	0.78^***^	–		
4. Psychopathology_Wave 1_	−0.44^***^	−0.33^***^	−0.27^***^	–	
5. Psychopathology_Wave 2_	−0.32^***^	−0.47^***^	−0.32^***^	0.65^***^	–
6. Psychopathology_Wave 3_	−0.32^***^	−0.41^***^	−0.51^***^	0.54^***^	0.70^***^

^*^p < 0.05, ^**^p < 0.01,

*** *p* < 0.001.

Inspection of the MZ and DZ correlation matrices gives some first indications of genetic and environmental sources of variance within, and covariance between, psychopathology and personality. While all cross-twin within-trait and cross-twin cross-trait correlations are presented in Supplementary Table S2, the general pattern will be summarized in the following. All DZ correlations were about half the size of the MZ correlations, indicating genetic influences with negligible influence of shared environmental factors on individual differences in psychopathology and personality, as well as on the covariance between them. The difference between the MZ correlations and the phenotypic correlations indicates the degree of non-shared environmental influences, which include all influences that make twins different on a trait, including measurement error. The size and significance of the various genetic and environmental influences were further tested in biometrical analyses.

### Genetic and environmental variance in psychopathology and personality

Trivariate Cholesky decomposition models were fitted to estimate genetic and environmental contributions to variance in the psychopathology and personality factors. Consistent with the pattern of twin correlations, the AE models provided better fit compared to the full ACE models, as indicated by the lowest AIC and BIC values (see Supplementary Table S3). Heritability and non-shared environmental variance from the univariate AE models (for details, see Supplementary Table S4) suggested that genetic influences accounted for a substantial proportion of individual differences in both psychopathology (a^2^: 0.54–0.59) and personality (a^2^: 0.49–0.53) at all measurement waves. The remaining variance, not accounted for by additive genetic influences, was attributed to non-shared environmental influences, including measurement error.

### Etiological relationship between psychopathology and personality

**Figure 2:**
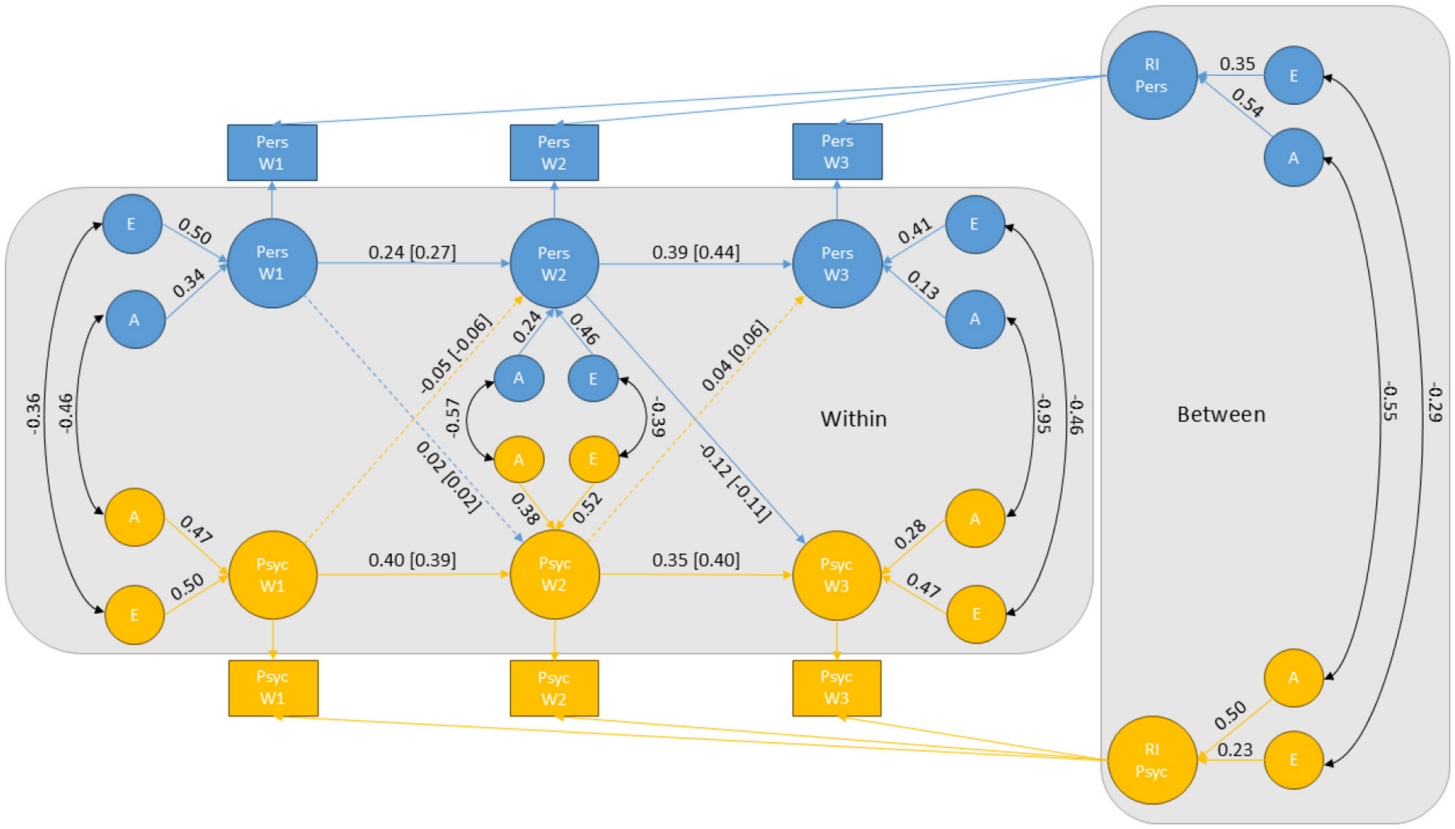
Genetically informative RI-CLPM for the relationship between psychopathology and personality. Standardized coefficients for the autoregressive and cross-lagged paths are provided in square brackets to enable comparison. Non-significant paths are indicated by dashed lines. Pers, personality; Psyc, psychopathology; RI, random intercept; A, additive genetic factors; E, non-shared environmental factors; W1, W2, and W3, measurement wave 1, 2, and 3, respectively.

To address our first research question (Q1: to what extent does change in personality predict change in psychopathology, and vice versa?), a genetically informative RI-CLPM was fitted to data. Based on the variance decompositions of psychopathology and personality, which indicated no influence of shared environmental factors, we included only A and E influences in the cross-lagged panel model. The RI-CLPM showed good absolute fit, with CFI = 0.996, TLI = 0.997 and RMSEA = 0.012. Unstandardized parameter estimates derived from the RI-CLPM for the relationship between general psychopathology and personality are displayed in Figure 2.

All cross-lagged paths were negligible in magnitude, indicating that changes in personality did not seem to predict changes in psychopathology, or vice versa. Although the cross-lagged path from personality at wave 2 to psychopathology at wave 3 was statistically significant, the effect was weak in strength, as indicated by the standardized estimate showing that only 1% of the fluctuations in psychopathology at wave 3 were explained by fluctuations in personality at wave 2.

The RI-CLPM also provides insight into our second research question (Q2: How stable are psychopathology and personality over time, and to what extent is this stability influenced by common genetic and environmental factors?). Table 3 presents the proportion of variance in psychopathology and personality accounted for by the random intercepts (i.e., time-invariant stability), as well as the proportion of genetic and environmental influences contributing to the stability. Equations used to calculate these proportions are described in detail in Skaug et al. (2024). Both psychopathology and personality showed a relatively high level of stability, with the random intercepts explaining between 53 and 64% of the total variance in personality and between 38 and 45% of the total variance in psychopathology. Genetic influences explained most of the time-invariant variance (i.e., 70% for personality and 83% for psychopathology). The genetic correlation between the random intercepts was −0.55, suggesting that some of the same genetic factors influence the stability of both psychopathology and personality.

**Table 3:** Time-invariant stability in the constructs.

**Measure**	**Proportion of variance explained by the random intercepts**			**Proportion of variance in the random intercepts due to genetic and environmental influences**	
	**Wave 1**	**Wave 2**	**Wave 3**	**A**	**E**
Personality	53%	58%	64%	70%	30%
Psychopathology	39%	38%	45%	83%	17%

A, additive genetic influences; E, non-shared environmental influences.

To deepen our understanding of the relationship between stable traits of psychopathology and personality, we fitted bivariate Cholesky models to aggregated measures of psychopathology and personality, capturing the shared variance of these constructs across all measurement waves. To create aggregated measures of psychopathology and personality, we employed factor analyses. This approach allowed us to combine the measures of psychopathology and personality across the three measurement waves into two single, aggregated scores—one for personality and one for psychopathology—that capture the stability (i.e., shared variance) of these constructs over time. Next, we fitted bivariate Cholesky models to these measures and investigated the extent of shared genetic variance underlying stable traits of psychopathology and personality. Results from these models are provided in Table 4. For the composite measure of personality, 26% of the genetic variance was shared with psychopathology. For the composite score of psychopathology, 27% of the genetic variance was shared with personality.

**Table 4:** Heritability (a^2^) and proportion of non-shared environmental variance (e^2^) underlying stable traits of psychopathology and personality, and the proportions of shared and unique genetic and environmental variance.

**Measure**	**Genetic effects**			**Non-shared environmental effects**		
	**a** ^2^	**% Shared** ^a^	**% Unique**	**e** ^2^	**% Shared** ^a^	**% Unique**
Personality	0.57	26.3	73.7	0.43	14.0	86.0
Psychopathology	0.63	27.0	73.0	0.37	13.5	86.5

a For personality, “% shared” reflects the percentage of variance shared with psychopathology. For psychopathology, “% shared” reflects the percentage of variance shared with personality.

## Discussion

This is the first study to our knowledge to investigate the etiological relationship between the general factors of psychopathology and personality from adolescence into young adulthood using a genetically informative cross-lagged design.

The primary research question, analyzed using a random intercept cross-lagged panel model, examined the extent to which change in one general factor predicted change in the other over time. The results showed that the cross-lagged influences between psychopathology and personality were negligible, indicating that changes in personality do not lead to changes in psychopathology, or vice versa.

These findings suggest that psychopathology and personality tend to develop independently of each other from adolescence into young adulthood. The lack of similar studies limits direct comparisons of these results. However, a recent study by Etkin et al. (2022) also reported non-significant cross-lagged paths over a 1-year period in adolescence between general personality and general psychopathology. Regarding the theoretical pathoplasticity, scar, and complication models mentioned in the introduction, our study's results did not support a direct causal relationship between psychopathology and personality. Specifically, changes in personality did not appear to alter levels of psychopathology, nor did changes in psychopathology seem to influence one's personality.

Considering the second research question, the results from the cross-lagged model indicated that both psychopathology and personality were relatively stable traits throughout adolescence and into young adulthood. These results concur well with the results reported in studies within as well as across mental disorders (Copeland et al., 2021; Plana-Ripoll et al., 2019; Rutter et al., 2006) and within and across personality traits across the life span, even during the “sturm und drang” years of adolescence (Lucas and Donnellan, 2011; Roberts and Delvecchio, 2000; Roberts et al., 2006). In their study on the stability and change in psychopathology over the lifespan, Caspi et al. (2020) observed significant fluctuations in single diagnoses but substantial heterotypic (cross-diagnostic) stability that accumulated from childhood and early adolescence onward. They also found a good fit for a general *p* factor, where higher scores on this factor indicated greater severity, characterized by earlier onset, and heterotypic stability. Similarly, in the domain of personality, although not specifically examining the general factor level, a comprehensive meta-analysis by Ferguson (2010) demonstrated high stability of both normal and disordered personality traits from age 14 through adulthood, encompassing cross-cultural samples and individuals in clinical treatment.

The variance decomposition of the stable (time-invariant) variance revealed that the stability of both traits was primarily attributable to additive genetic influences. Substantial genetic contributions to general psychopathology have been consistently documented across developmental stages, from early childhood to adulthood (Allegrini et al., 2020; Avinun et al., 2022; Riglin et al., 2020; Waldman et al., 2016). Similarly, various measures across different samples have indicated significant genetic influence on the general personality factor (Gigantesco et al., 2021; Loehlin and Martin, 2011; Rushton et al., 2009; Van Der Linden et al., 2022; Veselka et al., 2012, 2009).

While the results indicated considerable additive genetic influences contributing to the stability of the two general traits, only about one-quarter of the additive genetic variance in one trait was shared with the variance in the other. To our knowledge, the extent of shared genetic variance underlying the stability of psychopathology and personality has not previously been explored with both traits measured at the general factor level. However, substantial shared genetic influences between psychopathology and personality have been reported at the specific disorder/factor level, alongside specific genetic variance unique to each domain (Gjerde et al., 2023; Hansell et al., 2012; Hettema et al., 2006; Rosenström et al., 2019). These findings suggest that although psychopathology and personality share a significant portion of genetic variance—implying that one may serve as a potential risk or vulnerability factor for the other, as proposed by Ormel et al. (2013)—the majority of the genetic variance in personality remains distinct from that in psychopathology, and vice versa.

These results question traditional models of a dynamic causal relationship between psychopathology and personality, also emphasizing the need for empirical evidence in modeling connections within mental health. Comprehensive interdisciplinary empirically based initiatives such as the HiTOP represent promising avenues for future research. Within this framework, personality—especially trait neuroticism and negative emotionality—has been proposed to fit within the hierarchical structure of psychopathology, possibly as a supraspectrum for internalizing disorders (Lahey et al., 2017), or even as the overarching p-factor of all psychopathology (Brandes and Tackett, 2019). However, our findings advise caution against fully integrating these two aspects of mental functioning. Although psychopathology and personality are phenotypically and genetically related, they are distinct enough to warrant different conceptual statuses. The seemingly paradoxical persistence of genes that elevate the risk for mental disorders alongside those influencing adaptive personality traits may be elucidated through evolutionary genetics. Trait variation allows individuals to offer diverse strengths and strategies to social groups, and through processes including mutation-selection and balancing selection (Keller, 2008), survival is boosted in changing environments and against threats. Genetic diversity is sustained because different traits are advantageous in different contexts, enabling both personality and psychopathological traits to persist in the gene pool.

### Strengths and limitations

A major strength of the present study is the three-wave longitudinal design and behaviorally genetically informative approach, which together provide a unique possibility to study the etiological nature of association between the two general factors of psychopathology and personality. Studying the transition from adolescence to young adulthood provides a valuable developmental perspective to the research field. While psychopathology and personality demonstrate high stability in adult samples, this developmental phase offers a prime opportunity to study change, given that many mental disorders emerge in, and personality development is still in progress, during this period. Moreover, the large population-based sample using seven national cohorts of twins increases the likelihood that the findings can be generalized. While attrition may typically pose a threat to the generalizability of findings in longitudinal designs, analyses of recruitment and dropout within the twin sample used in this study revealed that attrition did not impact the heritability estimates (Torgersen and Waaktaar, 2019). Simulation studies have also indicated that attrition primarily impacts the validity of prevalence estimates, rather than the validity of estimates of associations between variables, which is the focus of the present study (Gustavson et al., 2012; Knudsen et al., 2010). Furthermore, the data, based on adolescent self-report, may be preferable for measuring internalizing problems and norm- and law-breaking behavior that parents and teachers of young people might not be aware of.

Certain specifics regarding the modeling of the general factors warrant consideration. General factors can be generated and modeled using various methods, each with its own advantages and challenges (Bornovalova et al., 2020). In this study, we specifically focused on a general psychopathology factor and a general personality factor. It was not within the scope of this article to test various factor structures. Rather, our aim was to examine the etiological relationship between general psychopathology and personality. Therefore, while our findings contribute to the understanding of the relationship between these general factors, further research is needed to explore alternative factor structures and to determine whether the results from such analyses are similar to or differ from our findings.

To accurately measure change in the factors over time, it is necessary to exclude variation arising from shifts in the relative influence of the measures within each factor across different time points. In the present study, this was addressed by keeping the factor loadings equal across waves, assuming the structure of personality traits (and psychopathology) remains constant over time. The issue of conceptual consistency of general factors is open to extensive exploration and discussion (Brandt et al., 2020; Forbes et al., 2021; Gluschkoff et al., 2019; Lucas and Donnellan, 2011; Scopel Hoffmann et al., 2022). In a recent decade-long research review on the general factor of psychopathology, Caspi et al. (2024) caution against becoming overly fixated on the technicalities of factor analyses and measurement models, as different approaches tend to perform similarly well given equivalent sample sizes and content. The practical approach chosen here allowed for attributing change within the general factors over time to actual change rather than to varying influences of individual traits. This method also simplified the model and controlled for potential measurement error, thereby enhancing the validity and interpretability of our results.

By definition, any general factor consist of the contributing indicators, and low factor loadings may indicate that the factor may not really represent the idea of a global factor well (Lahey et al., 2015; Markon, 2019). The resulting general factors in the present study accounted for 44% of the variance in the personality measures and 43% of the variance in the psychopathology measures, with factor loadings generally falling within the moderate to high range. Other studies have also reported similar levels of explained variance in the general factor of psychopathology (*p*) during adolescence (Harris et al., 2024). Similarly, Rushton and Irwing (2008) and Van Der Linden et al. (2010) found comparable percentages of explained variance in the General Factor of Personality (GFP) across several meta-analyses.

### Conclusion

This study is the first to investigate the etiological relationship between the general factors of psychopathology and personality from adolescence to young adulthood using a longitudinal twin design. The results indicated that change in personality does not predict change in psychopathology, and vice versa. There was considerable stability within both traits throughout adolescence, predominantly explained by genetic influences. Although a significant portion of this stability was due to genetic influences shared between psychopathology and personality, most of the genetic variance was unique to each trait. Thus, general psychopathology and general personality seem to develop independently during adolescence and into young adulthood, with considerable stability in both traits largely driven by genetic influences specific to each trait. Shared genetic etiology in the stability of both traits suggests that psychopathology and personality may mutually serve as potential risk factors for each other during this developmental period. The idea of influencing change in one trait by targeting the other is not supported in this study. However, such hypotheses would need to be tested within an intervention design to draw conclusions about direct causation.

## Data Availability

The datasets presented in this article are not readily available because data collection received preapproval in 2005 from the Norwegian Data Protection Authority, mandating a 20-year period of individual data protection followed by either data deletion or anonymization. Anonymized data will be available upon request after 2025. Requests to access the datasets should be directed to trine.waaktaar@psykologi.uio.no.

## References

[B1] Akaike H. (1987). Factor analysis and AIC. Psychometrika 52, 317–332. 10.1007/BF02294359

[B2] Allegrini A. G. Cheesman R. Rimfeld K. Selzam S. Pingault J. B. Eley T. C.. (2020). The p factor: genetic analyses support a general dimension of psychopathology in childhood and adolescence. J. Child Psychol. Psychiatry 61, 30–39. 10.1111/jcpp.13113 31541466 PMC6906245

[B3] Ask H. Torgersen S. Seglem K. B. Waaktaar T. (2014). Genetic and environmental causes of variation in adolescent anxiety symptoms: a multiple-rater twin study. J. Anxiety Disord. 28, 363–371. 10.1016/j.janxdis.2014.04.003 24793742

[B4] Ask H. Waaktaar T. Seglem K. B. Torgersen S. (2016). Common etiological sources of anxiety, depression, and somatic complaints in adolescents: a multiple rater twin study. J. Abnorm. Child Psychol. 44, 101–114. 10.1007/s10802-015-9977-y 25619928

[B5] Avinun R. Knafo-Noam A. Israel S. (2022). The general psychopathology factor from early to middle childhood: longitudinal genetic and risk analyses. J. Psychopathol. Clin. Sci. 131, 705–715. 10.1037/abn0000780 36222626

[B6] Birmaher B. Khetarpal S. Brent D. Cully M. Balach L. Kaufman J.. (1997). The screen for child anxiety related emotional disorders (SCARED): scale construction and psychometric characteristics. J. Am. Acad. Child Adolesc. Psychiatry 36, 545–553. 10.1097/00004583-199704000-00018 9100430

[B7] Bleidorn W. Schwaba T. Zheng A. Hopwood C. J. Sosa S. S. Roberts B. W.. (2022). Personality stability and change: a meta-analysis of longitudinal studies. Psychol. Bull. 148, 588–619. 10.1037/bul0000365 35834197

[B8] Bornovalova M. A. Choate A. M. Fatimah H. Petersen K. J. Wiernik B. M. (2020). Appropriate use of bifactor analysis in psychopathology research: appreciating benefits and limitations. Biol. Psychiatry 88, 18–27. 10.1016/j.biopsych.2020.01.013 32199605 PMC10586518

[B9] Boyette L.-L. Korver-Nieberg N. Verweij K. Meijer C. Dingemans P. Cahn W.. (2013). Associations between the Five-Factor Model personality traits and psychotic experiences in patients with psychotic disorders, their siblings and controls. Psychiatry Res. 210, 491–497. 10.1016/j.psychres.2013.06.040 23890697

[B10] Brandes C. M. Tackett J. L. (2019). Contextualizing neuroticism in the hierarchical taxonomy of psychopathology. J. Res. Pers. 81, 238–245. 10.1016/j.jrp.2019.06.007

[B11] Brandt N. D. Becker M. Tetzner J. Brunner M. Kuhl P. Maaz K. (2020). Personality across the lifespan: exploring measurement invariance of a Short Big Five Inventory from ages 11 to 84. Eur. J. Psychol. Assess. 36, 162–173. 10.1027/1015-5759/a000490

[B12] Briley D. A. Livengood J. Derringer J. (2018). Behaviour genetic frameworks of causal reasoning for personality psychology. Eur. J. Pers. 32, 202–220. 10.1002/per.2153

[B13] Caspi A. Houts R. M. Ambler A. Danese A. Elliott M. L. Hariri A.. (2020). Longitudinal assessment of mental health disorders and comorbidities across 4 decades among participants in the Dunedin birth cohort study. JAMA Netw. Open 3:e203221. 10.1001/jamanetworkopen.2020.3221 32315069 PMC7175086

[B14] Caspi A. Houts R. M. Belsky D. W. Goldman-Mellor S. J. Harrington H. Israel S.. (2014). The p factor: one general psychopathology factor in the structure of psychiatric disorders? Clin. Psychol. Sci. 2, 119–137. 10.1177/2167702613497473 25360393 PMC4209412

[B15] Caspi A. Houts R. M. Fisher H. L. Danese A. Moffitt T. E. (2024). The general factor of psychopathology (p): choosing among competing models and interpreting p. Clin. Psychol. Sci. 12, 53–82. 10.1177/21677026221147872 38236494 PMC10794018

[B16] Caspi A. Moffitt T. E. (2018). All for one and one for all: mental disorders in one dimension. Am. J. Psychiatry 175, 831–844. 10.1176/appi.ajp.2018.17121383 29621902 PMC6120790

[B17] Cicchetti D. Rogosch F. A. (2002). A developmental psychopathology perspective on adolescence. J. Consult. Clin. Psychol. 70:6. 10.1037//0022-006X.70.1.6 11860057

[B18] Copeland W. E. Alaie I. Jonsson U. Shanahan L. (2021). Associations of childhood and adolescent depression with adult psychiatric and functional outcomes. J. Am. Acad. Child Adolesc. Psychiatry 60, 604–611. 10.1016/j.jaac.2020.07.895 32758528 PMC8051642

[B19] Costa P. T. Mccrae R. R. (1992). The five-factor model of personality and its relevance to personality disorders. J. Pers. Disord. 6, 343–359. 10.1521/pedi.1992.6.4.343

[B20] Dalsgaard S. Thorsteinsson E. Trabjerg B. B. Schullehner J. Plana-Ripoll O. Brikell I.. (2020). Incidence rates and cumulative incidences of the full spectrum of diagnosed mental disorders in childhood and adolescence. JAMA Psychiatry 77, 155–164. 10.1001/jamapsychiatry.2019.3523 31746968 PMC6902162

[B21] Davies S. E. Connelly B. S. Ones D. S. Birkland A. S. (2015). The general factor of personality: the “Big One,” a self-evaluative trait, or a methodological gnat that won't go away? Pers. Individ. Dif. 81, 13–22. 10.1016/j.paid.2015.01.006

[B22] Durbin C. E. Hicks B. M. (2014). Personality and psychopathology: a stagnant field in need of development. Eur. J. Pers. 28, 362–386. 10.1002/per.1962 25544802 PMC4276423

[B23] Erdle S. Rushton J. P. (2011). Does self-esteem or social desirability account for a general factor of personality (GFP) in the Big Five? Pers. Individ. Dif. 50, 1152–1154. 10.1016/j.paid.2010.12.038

[B24] Etkin P. Ibáñez M. I. Ortet G. Mezquita L. (2022). Longitudinal associations between the five-factor model of personality and the bi-factor model of psychopathology: continuity, pathoplasty and complication effects in adolescents. J. Psychopathol. Behav. Assess. 44, 405–417. 10.1007/s10862-021-09903-1

[B25] Ferguson C. J. (2010). A meta-analysis of normal and disordered personality across the life span. J. Pers. Soc. Psychol. 98, 659–667. 10.1037/a0018770 20307136

[B26] Forbes M. K. Greene A. L. Levin-Aspenson H. F. Watts A. L. Hallquist M. Lahey B. B.. (2021). Three recommendations based on a comparison of the reliability and validity of the predominant models used in research on the empirical structure of psychopathology. J. Abnorm. Psychol. 130, 297–317. 10.1037/abn0000533 33539117

[B27] Fried E. I. Greene A. L. Eaton N. R. (2021). The p factor is the sum of its parts, for now. World Psychiatry 20, 69. 10.1002/wps.20814 33432741 PMC7801826

[B28] Garber J. Walker L. S. Zeman J. (1991). Somatization symptoms in a community sample of children and adolescents: further validation of the Children's Somatization Inventory. Psychol. Assess. J. Consult. Clin. Psychol. 3:588. 10.1037/1040-3590.3.4.588

[B29] Garner D. M. (2004). Eating Disorder Inventory-3 (EDI-3). Professional Manual, Vol 1. Odessa, FL: Psychological Assessment Resources.

[B30] Gigantesco A. Fagnani C. Alessandri G. Carluccio E. Stazi M. A. Medda E. (2021). Genetic and environmental architecture of five factor model and super-factors: an Italian Twin Study. Span. J. Psychol. 25:e2. 10.1017/SJP.2021.48 34957942

[B31] Gjerde L. C. Eilertsen E. M. Mcadams T. A. Cheesman R. Moffitt T. E. Caspi A.. (2023). The p factor of psychopathology and personality in middle childhood: genetic and gestational risk factors. Psychol. Med. 53, 4275–4285. 10.1017/S0033291723000077 36762420 PMC10317823

[B32] Gluschkoff K. Jokela M. Rosenström T. (2019). The general psychopathology factor: structural stability and generalizability to within-individual changes. Front Psychiatry 10:594. 10.3389/fpsyt.2019.00594 31543833 PMC6728891

[B33] Goodman R. (1997). The strengths and difficulties questionnaire: a research note. J. Child Psychol. Psychiatry 38, 581–586. 10.1111/j.1469-7610.1997.tb01545.x 9255702

[B34] Goodman R. (2001). Psychometric properties of the strengths and difficulties questionnaire. J. Am. Acad. Child Adolesc. Psychiatry 40, 1337–1345. 10.1097/00004583-200111000-00015 11699809

[B35] Griffith J. W. Zinbarg R. E. Craske M. G. Mineka S. Rose R. D. Waters A. M.. (2010). Neuroticism as a common dimension in the internalizing disorders. Psychol. Med. 40, 1125–1136. 10.1017/S0033291709991449 19903363 PMC2882529

[B36] Gustavson K. Von Soest T. Karevold E. Røysamb E. (2012). Attrition and generalizability in longitudinal studies: findings from a 15-year population-based study and a Monte Carlo simulation study. BMC Public Health 12:918. 10.1186/1471-2458-12-918 23107281 PMC3503744

[B37] Hamaker E. L. Kuiper R. M. Grasman R. P. P. P. (2015). A critique of the cross-lagged panel model. Psychol. Methods 20, 102–116. 10.1037/a0038889 25822208

[B38] Hansell N. K. Wright M. J. Medland S. E. Davenport T. A. Wray N. R. Martin N. G.. (2012). Genetic co-morbidity between neuroticism, anxiety/depression and somatic distress in a population sample of adolescent and young adult twins. Psychol. Med. 42, 1249–1260. 10.1017/S0033291711002431 22051348

[B39] Harris J. L. Swanson B. Petersen I. T. (2024). A developmentally informed systematic review and meta-analysis of the strength of general psychopathology in childhood and adolescence. Clin. Child Fam. Psychol. Rev. 27, 130–164. 10.1007/s10567-023-00464-1 38112921 PMC10938301

[B40] Haslam N. Mcgrath M. J. Viechtbauer W. Kuppens P. (2020). Dimensions over categories: a meta-analysis of taxometric research. Psychol. Med. 50, 1418–1432. 10.1017/S003329172000183X 32493520

[B41] Hengartner M. P. Ajdacic-Gross V. Wyss C. Angst J. Rössler W. (2016). Relationship between personality and psychopathology in a longitudinal community study: a test of the predisposition model. Psychol. Med. 46, 1693–1705. 10.1017/S0033291716000210 26979285

[B42] Hettema J. M. Neale M. C. Myers J. M. Prescott C. A. Kendler K. S. (2006). A population-based twin study of the relationship between neuroticism and internalizing disorders. Am. J. Psychiatry 163, 857–864. 10.1176/ajp.2006.163.5.857 16648327

[B43] Hu L.-T. Bentler P. M. (1999). Cutoff criteria for fit indexes in covariance structure analysis: conventional criteria versus new alternatives. Struct. Equat. Model. Multidiscipl. J. 6, 1–55. 10.1080/10705519909540118

[B44] John O. P. Angleitner A. Ostendorf F. (1988). The lexical approach to personality: a historical review of trait taxonomic research. Eur. J. Pers. 2, 171–203. 10.1002/per.2410020302

[B45] Jylhä P. Melartin T. Isometsä E. (2009). Relationships of neuroticism and extraversion with axis I and II comorbidity among patients with DSM-IV major depressive disorder. J. Affect. Disord. 114, 110–121. 10.1016/j.jad.2008.06.011 18687471

[B46] Kandler C. Waaktaar T. Mõttus R. Riemann R. Torgersen S. (2019). Unravelling the interplay between genetic and environmental contributions in the unfolding of personality differences from early adolescence to young adulthood. Eur. J. Pers. 33, 221–244. 10.1002/per.2189

[B47] Keller M. C. (2008). The evolutionary persistence of genes that increase mental disorders risk. Curr. Dir. Psychol. Sci. 17, 395–399. 10.1111/j.1467-8721.2008.00613.x

[B48] Kendler K. S. Neale M. C. Kessler R. C. Heath A. C. Eaves L. J. (1993). A longitudinal twin study of personality and major depression in women. Arch. Gen. Psychiatry 50, 853–862. 10.1001/archpsyc.1993.01820230023002 8215811

[B49] Kessler R. C. Chiu W. T. Demler O. Merikangas K. R. Walters E. E. (2005). Prevalence, severity, and comorbidity of 12-month DSM-IV disorders in the National Comorbidity Survey Replication. Arch. Gen. Psychiatry 62, 617–627. 10.1001/archpsyc.62.6.617 15939839 PMC2847357

[B50] Kim-Cohen J. Caspi A. Moffitt T. E. Harrington H. Milne B. J. Poulton R. (2003). Prior juvenile diagnoses in adults with mental disorder: developmental follow-back of a prospective-longitudinal cohort. Arch. Gen. Psychiatry 60, 709–717. 10.1001/archpsyc.60.7.709 12860775

[B51] Knight J. R. Sherritt L. Shrier L. A. Harris S. K. Chang G. (2002). Validity of the CRAFFT substance abuse screening test among adolescent clinic patients. Arch. Pediatr. Adolesc. Med. 156, 607–614. 10.1001/archpedi.156.6.607 12038895

[B52] Knight J. R. Shrier L. A. Bravender T. D. Farrell M. Vander Bilt J. Shaffer H. J. (1999). A new brief screen for adolescent substance abuse. Arch. Pediatr. Adolesc. Med. 153, 591–596. 10.1001/archpedi.153.6.591 10357299

[B53] Knudsen A. K. Hotopf M. Skogen J. C. Øverland S. Mykletun A. (2010). The health status of nonparticipants in a population-based health study: the Hordaland Health Study. Am. J. Epidemiol. 172, 1306–1314. 10.1093/aje/kwq257 20843863

[B54] Kotov R. Gamez W. Schmidt F. Watson D. (2010). Linking “big” personality traits to anxiety, depressive, and substance use disorders: a meta-analysis. Psychol. Bull. 136, 768–821. 10.1037/a0020327 20804236

[B55] Kotov R. Krueger R. F. Watson D. Cicero D. C. Conway C. C. Deyoung C. G.. (2021). The hierarchical taxonomy of psychopathology (hitop): a quantitative nosology based on consensus of evidence. Annu. Rev. Clin. Psychol. 17, 83–108. 10.1146/annurev-clinpsy-081219-093304 33577350

[B56] Krueger R. F. Tackett J. L. (2003). Personality and psychopathology: working toward the bigger picture. J. Pers. Disord. 17, 109–128. 10.1521/pedi.17.2.109.23986 12755325

[B57] Lahey B. B. Applegate B. Hakes J. K. Zald D. H. Hariri A. R. Rathouz P. J. (2012). Is there a general factor of prevalent psychopathology during adulthood? J. Abnorm. Psychol. 121, 971–977. 10.1037/a0028355 22845652 PMC4134439

[B58] Lahey B. B. Applegate B. Waldman I. D. Loft J. D. Hankin B. L. Rick J. (2004). The structure of child and adolescent psychopathology: generating new hypotheses. J. Abnorm. Psychol. 113, 358. 10.1037/0021-843X.113.3.358 15311983

[B59] Lahey B. B. Krueger R. F. Rathouz P. J. Waldman I. D. Zald D. H. (2017). A hierarchical causal taxonomy of psychopathology across the life span. Psychol. Bull. 143, 142–186. 10.1037/bul0000069 28004947 PMC5269437

[B60] Lahey B. B. Rathouz P. J. Keenan K. Stepp S. D. Loeber R. Hipwell A. E. (2015). Criterion validity of the general factor of psychopathology in a prospective study of girls. J. Child Psychol. Psychiatry 56, 415–422. 10.1111/jcpp.12300 25052460 PMC4435546

[B61] Leblanc M. Tremblay R. (1988). A study of factors associated with the stability of hidden delinquency. Int. J. Adolesc. Youth 1, 269–291. 10.1080/02673843.1988.9747643

[B62] Littlefield A. K. Lane S. P. Gette J. A. Watts A. L. Sher K. J. (2021). The “Big Everything”: integrating and investigating dimensional models of psychopathology, personality, personality pathology, and cognitive functioning. Persl. Disord. 12, 103–114. 10.1037/per0000457 32915005 PMC8318803

[B63] Loehlin J. C. Martin N. G. (2011). The general factor of personality: questions and elaborations. J. Res. Pers. 45, 44–49. 10.1016/j.jrp.2010.11.008

[B64] Lucas R. E. Donnellan M. B. (2011). Personality development across the life span: longitudinal analyses with a national sample from Germany. J. Pers. Soc. Psychol. 101, 847–861. 10.1037/a0024298 21707197

[B65] Lüdtke O. Trautwein U. Husemann N. (2009). Goal and personality trait development in a transitional period: assessing change and stability in personality development. Pers. Soc. Psychol. Bull. 35, 428–441. 10.1177/0146167208329215 19144768

[B66] Lynch S. J. Sunderland M. Newton N. C. Chapman C. (2021). A systematic review of transdiagnostic risk and protective factors for general and specific psychopathology in young people. Clin. Psychol. Rev. 87, 102036. 10.1016/j.cpr.2021.102036 33992846

[B67] Malouff J. M. Thorsteinsson E. B. Schutte N. S. (2005). The relationship between the five-factor model of personality and symptoms of clinical disorders: a meta-analysis. J. Psychopathol. Behav. Assess. 27, 101–114. 10.1007/s10862-005-5384-y

[B68] Markon K. E. (2019). Bifactor and hierarchical models: specification, inference, and interpretation. Annu. Rev. Clin. Psychol. 15, 51–69. 10.1146/annurev-clinpsy-050718-095522 30649927

[B69] Mccrae R. R. Costa P. T. Jr. (2008). The SAGE Handbook of Personality Theory and Assessment: Volume 1 — Personality Theories and Models. London: SAGE Publications Ltd. 10.4135/9781849200462.n1

[B70] Mcelroy E. Belsky J. Carragher N. Fearon P. Patalay P. (2018). Developmental stability of general and specific factors of psychopathology from early childhood to adolescence: dynamic mutualism or p-differentiation? J. Child Psychol. Psychiatry 59, 667–675. 10.1111/jcpp.12849 29197107 PMC6001631

[B71] Mervielde I. De Fruyt F. (1999). “Construction of the Hierarchical Personality Inventory for Children (HiPIC). I: Mervielde, I., Deary, F., De Fruyt, F. and Ostendorf, F. (red.) Personality Psychology in Europe,” in Proceedings of the Eight European Conference on Personality Psychology (Tilburg, Netherlands: Tilburg University Press). 10.1037/t20166-000

[B72] Murray A. L. Eisner M. Ribeaud D. (2016). The development of the general factor of psychopathology ‘p Factor' through childhood and adolescence. J. Abnorm. Child Psychol. 44, 1573–1586. 10.1007/s10802-016-0132-1 26846993

[B73] Musek J. (2007). A general factor of personality: evidence for the Big One in the five-factor model. J. Res. Pers. 41, 1213–1233. 10.1016/j.jrp.2007.02.003

[B74] Neale M. C. Hunter M. D. Pritikin J. N. Zahery M. Brick T. R. Kirkpatrick R. M.. (2016). OpenMx 2.0: extended structural equation and statistical modeling. Psychometrika 81, 535–549. 10.1007/s11336-014-9435-8 25622929 PMC4516707

[B75] Neale M. C. Maes H. H. M. (2004). Methodology for Genetic Studies of Twins and Families. Dordrecht: Kluwer Academic Publishers B.V.

[B76] Nicolson R. Brookner F. B. Lenane M. Gochman P. Ingraham L. J. Egan M. F.. (2003). Parental schizophrenia spectrum disorders in childhood-onset and adult-onset schizophrenia. Am. J. Psychiatry 160, 490–495. 10.1176/appi.ajp.160.3.490 12611830

[B77] Ormel J. Jeronimus B. F. Kotov R. Riese H. Bos E. H. Hankin B.. (2013). Neuroticism and common mental disorders: meaning and utility of a complex relationship. Clin. Psychol. Rev. 33, 686–697. 10.1016/j.cpr.2013.04.003 23702592 PMC4382368

[B78] Ormel J. Raven D. Van Oort F. Hartman C. A. Reijneveld S. A. Veenstra R.. (2015). Mental health in Dutch adolescents: a TRAILS report on prevalence, severity, age of onset, continuity and co-morbidity of DSM disorders. Psychol. Med. 45, 345–360. 10.1017/S0033291714001469 25066533

[B79] Paus T. Keshavan M. Giedd J. N. (2008). Why do many psychiatric disorders emerge during adolescence? Nat. Rev. Neurosci. 9, 947–957. 10.1038/nrn2513 19002191 PMC2762785

[B80] Plana-Ripoll O. Pedersen C. B. Holtz Y. Benros M. E. Dalsgaard S. De Jonge P.. (2019). Exploring comorbidity within mental disorders among a Danish national population. JAMA Psychiatry 76, 259–270. 10.1001/jamapsychiatry.2018.3658 30649197 PMC6439836

[B81] Plomin R. Haworth C. M. A. Davis O. S. P. (2009). Common disorders are quantitative traits. Nat. Rev. Genet. 10, 872–878. 10.1038/nrg2670 19859063

[B82] Radloff L. S. (1977). The CES-D scale: a self-report depression scale for research in the general population. Appl. Psychol. Meas. 1, 385–401. 10.1177/014662167700100306

[B83] Raftery A. E. (1995). Bayesian model selection in social research. Sociol. Methodol. 25, 111–163. 10.2307/271063

[B84] Riglin L. Thapar A. K. Leppert B. Martin J. Richards A. Anney R.. (2020). Using genetics to examine a general liability to childhood psychopathology. Behav. Genet. 50, 213–220. 10.1007/s10519-019-09985-4 31828458 PMC7355267

[B85] Ristić I. KneŽević G. Ristić D. I. Miljević C. Jerotić S. Marić N. P. (2023). Do people diagnosed with psychosis spectrum disorders share the same personality space as the general population? Big Five complemented by the proneness to psychotic-like experiences/behaviors. J. Pers. 91, 1381–1394. 10.1111/jopy.12814 36660808

[B86] Roberts B. W. Delvecchio W. F. (2000). The rank-order consistency of personality traits from childhood to old age: a quantitative review of longitudinal studies. Psychol. Bull. 126, 3–25. 10.1037//0033-2909.126.1.3 10668348

[B87] Roberts B. W. Walton K. E. Viechtbauer W. (2006). Patterns of mean-level change in personality traits across the life course: a meta-analysis of longitudinal studies. Psychol. Bull. 132, 1–25. 10.1037/0033-2909.132.1.1 16435954

[B88] Rosenström T. Gjerde L. C. Krueger R. F. Aggen S. H. Czajkowski N. O. Gillespie N. A.. (2019). Joint factorial structure of psychopathology and personality. Psychol. Med. 49, 2158–2167. 10.1017/S0033291718002982 30392478

[B89] Ruiz M. A. Pincus A. L. Schinka J. A. (2008). Externalizing pathology and the Five-Factor Model: a meta-analysis of personality traits associated with antisocial personality disorder, substance use disorder, and their co-occurrence. J. Pers. Disord. 22, 365–388. 10.1521/pedi.2008.22.4.365 18684050

[B90] Rushton J. P. Bons T. A. Ando J. Hur Y.-M. Irwing P. Vernon P. A.. (2009). A general factor of personality from multitrait–multimethod data and cross–national twins. Twin Res. Hum. Genet. 12, 356–365. 10.1375/twin.12.4.356 19653836

[B91] Rushton J. P. Irwing P. (2008). A General Factor of Personality (GFP) from two meta-analyses of the Big Five: Digman (1997) and Mount, Barrick, Scullen, and Rounds (2005). Pers. Individ. Dif. 45, 679–683. 10.1016/j.paid.2008.07.015

[B92] Rutter M. Kim-Cohen J. Maughan B. (2006). Continuities and discontinuities in psychopathology between childhood and adult life. J. Child Psychol. Psychiatry 47, 276–295. 10.1111/j.1469-7610.2006.01614.x 16492260

[B93] Schwaba T. Bleidorn W. (2018). Individual differences in personality change across the adult life span. J. Pers. 86, 450–464. 10.1111/jopy.12327 28509384

[B94] Scopel Hoffmann M. Moore T. M. Kvitko Axelrud L. Tottenham N. Zuo X.-N. Rohde L. A.. (2022). Reliability and validity of bifactor models of dimensional psychopathology in youth. J. Psychopathol. Clin. Sci. 131, 407–421. 10.1037/abn0000749 35511526 PMC9328119

[B95] Seglem K. B. Torgersen S. Ask H. Waaktaar T. (2015). Weak etiologic links between control and the externalizing behaviors delinquency and substance abuse in adolescence. Pers. Individ. Dif. 75, 179–184. 10.1016/j.paid.2014.11.036

[B96] Skaug E. Czajkowski N. O. Waaktaar T. Torgersen S. (2022a). Childhood trauma and borderline personality disorder traits: a discordant twin study. J. Psychopathol. Clin. Sci. 131, 365–374. 10.1037/abn0000755 35377676

[B97] Skaug E. Czajkowski N. O. Waaktaar T. Torgersen S. (2024). The longitudinal relationship between life events and loneliness in adolescence: a twin study. Dev. Psychol. 60, 966–977. 10.1037/dev0001692 38483481

[B98] Skaug E. Olavi Czajkowski N. Waaktaar T. Torgersen S. (2022b). The relationship between life events and sense of coherence in adolescence. A longitudinal twin study. J. Res. Pers. 99:104259. 10.1016/j.jrp.2022.104259

[B99] Snyder H. R. Young J. F. Hankin B. L. (2017). Strong homotypic continuity in common psychopathology-, internalizing-, and externalizing-specific factors over time in adolescents. Clin. Psychol. Sci. 5, 98–110. 10.1177/2167702616651076 28239532 PMC5320894

[B100] Spearman C. (1904). “General intelligence,” objectively determined and measured. Am. J. Psychol. 15, 201–292. 10.2307/1412107 14717630

[B101] Tackett J. L. (2006). Evaluating models of the personality-psychopathology relationship in children and adolescents. Clin. Psychol. Rev. 26, 584–599. 10.1016/j.cpr.2006.04.003 16820251

[B102] Torgersen S. (1979). The determination of twin zygosity by means of a mailed questionnaire. Acta Genet. Med. Gemellol. 28, 225–236. 10.1017/S0001566000009077 297423

[B103] Torgersen S. Waaktaar T. (2019). The Oslo University adolescent and young adult twin project: recruitment and attrition. Twin Res. Hum. Genet. 22, 641–646. 10.1017/thg.2019.51 31391138

[B104] Torgersen S. Waaktaar T. (2020). The Oslo University adolescent and young adult twin project: recruitment and attrition–corrigendum. Twin Res. Hum. Genet. 23, 358–358. 10.1017/thg.2020.88 33455602

[B105] Trull T. J. Sher K. J. (1994). Relationship between the five-factor model of personality and Axis I disorders in a nonclinical sample. J. Abnorm. Psychol. 103, 350–360. 10.1037//0021-843X.103.2.350 8040504

[B106] Uliaszek A. A. Hauner K. K. Zinbarg R. E. Craske M. G. Mineka S. Griffith J. W.. (2009). An examination of content overlap and disorder-specific predictions in the associations of neuroticism with anxiety and depression. J. Res. Pers. 43, 785–794. 10.1016/j.jrp.2009.05.009 20161016 PMC2748952

[B107] Van Buuren S. (2007). Multiple imputation of discrete and continuous data by fully conditional specification. Stat. Methods Med. Res. 16, 219–242. 10.1177/0962280206074463 17621469

[B108] Van Der Linden D. Dunkel C. S. De Zeeuw E. J. Wu P. Pelt D. H. M. (2022). The General Factor of Personality (GFP) and vocational interests: a test of social effectiveness at the behavioral and genetic level. J. Bus. Psychol. 37, 1017–1038. 10.1007/s10869-021-09779-8

[B109] Van Der Linden D. Te Nijenhuis J. Bakker A. B. (2010). The general factor of personality: a meta-analysis of Big Five intercorrelations and a criterion-related validity study. J. Res. Pers. 44, 315–327. 10.1016/j.jrp.2010.03.003

[B110] Veselka L. Schermer J. A. Just C. Hur Y.-M. Rushton J. P. Jeong H.-U.. (2012). Emotion and behavior: a general factor of personality from the EAS temperament survey and the strengths and difficulties questionnaire. Twin Res. Hum. Genet. 15, 668–671. 10.1017/thg.2012.21 22877833

[B111] Veselka L. Schermer J. A. Petrides K. V. Vernon P. A. (2009). Evidence for a heritable general factor of personality in two studies. Twin Res. Hum. Genet. 12, 254–260. 10.1375/twin.12.3.254 19456217

[B112] Waaktaar T. Torgersen S. (2012). Genetic and environmental causes of variation in trait resilience in young people. Behav. Genet. 42, 366–377. 10.1007/s10519-011-9519-5 22101958 PMC3350764

[B113] Waldman I. D. Poore H. E. Van Hulle C. Rathouz P. J. Lahey B. B. (2016). External validity of a hierarchical dimensional model of child and adolescent psychopathology: tests using confirmatory factor analyses and multivariate behavior genetic analyses. J. Abnorm. Psychol. 125, 1053–1066. 10.1037/abn0000183 27819467 PMC6810679

[B114] Waszczuk M. A. Waaktaar T. Eley T. C. Torgersen S. (2019). Etiological influences on continuity and co-occurrence of eating disorders symptoms across adolescence and emerging adulthood. Int. J. Eat. Disord. 52, 554–563. 10.1002/eat.23040 30729562

[B115] Watts A. L. Poore H. E. Waldman I. D. (2019). Riskier tests of the validity of the bifactor model of psychopathology. Clin. Psychol. Sci. 7, 1285–1303. 10.1177/2167702619855035

[B116] Widiger T. A. (2011). Personality and psychopathology. World Psychiatry 10, 103–106. 10.1002/j.2051-5545.2011.tb00024.x 21633679 PMC3104878

[B117] Widiger T. A. Trull T. J. (1992). Personality and psychopathology: an application of the five-factor model. J. Pers. 60, 363–393. 10.1111/j.1467-6494.1992.tb00977.x 1635047

[B118] Williams C. Daley D. Burnside E. Hammond-Rowley S. (2010). Does item overlap account for the relationship between trait emotional intelligence and psychopathology in preadolescents? Pers. Individ. Dif. 48, 867–871. 10.1016/j.paid.2010.02.006

[B119] Wilson S. Olino T. M. (2021). A developmental perspective on personality and psychopathology across the life span. J. Pers. 89, 915–932. 10.1111/jopy.12623 33550639 PMC10142293

[B120] Wright A. G. C. Krueger R. F. Hobbs M. J. Markon K. E. Eaton N. R. Slade T. (2013). The structure of psychopathology: toward an expanded quantitative empirical model. J. Abnorm. Psychol. 122, 281. 10.1037/a0030133 23067258 PMC3570590

[B121] Xia J. He Q. Li Y. Xie D. Zhu S. Chen J.. (2011). The relationship between neuroticism, major depressive disorder and comorbid disorders in Chinese women. J. Affect. Disord. 135, 100–105. 10.1016/j.jad.2011.06.053 21824661 PMC3220767

[B122] Zhou M. Larsson H. D'onofrio B. M. Landén M. Lichtenstein P. Pettersson E. (2023). Intergenerational transmission of psychiatric conditions and psychiatric, behavioral, and psychosocial outcomes in offspring. JAMA Netw. Open 6:e2348439. 10.1001/jamanetworkopen.2023.48439 38117496 PMC10733806

