# Supplementary material for: The etiological relationship between the general factors of psychopathology and personality; a longitudinal twin study from adolescence into young adulthood
Source: Front Psychol. 2025 Jul 8;16:1564305. doi: 10.3389/fpsyg.2025.1564305 (PMC12279783; doi:10.3389/fpsyg.2025.1564305)
Supplement: Supplementary file 1 [file Table_1.docx]

**Table S1**

*Descriptive Statistics*

|  | Wave 1 | |  | Wave 2 | |  | Wave 3 | |
| --- | --- | --- | --- | --- | --- | --- | --- | --- |
| Variable | *M* | *SD* |  | *M* | *SD* |  | *M* | *SD* |
| Neuroticism | 1.51 | 0.68 |  | 1.61 | 0.65 |  | 1.65 | 0.63 |
| Extraversion | 2.45 | 0.58 |  | 2.41 | 0.57 |  | 2.43 | 0.54 |
| Openness | 2.17 | 0.59 |  | 2.16 | 0.56 |  | 2.22 | 0.53 |
| Agreeableness | 2.80 | 0.55 |  | 2.78 | 0.51 |  | 2.82 | 0.47 |
| Conscientiousness | 2.41 | 0.60 |  | 2.37 | 0.57 |  | 2.44 | 0.52 |
| Depressive symptoms | 0.63 | 0.51 |  | 0.77 | 0.52 |  | 0.83 | 0.48 |
| Anxiety symptoms | 0.41 | 0.26 |  | 0.43 | 0.26 |  | 0.42 | 0.24 |
| Somatic complaints | 0.71 | 0.51 |  | 0.81 | 0.51 |  | 0.83 | 0.48 |
| Eating disorder symptoms | 0.83 | 0.88 |  | 1.04 | 0.85 |  | 1.18 | 0.81 |
| Delinquency | 0.13 | 0.19 |  | 0.19 | 0.18 |  | 0.18 | 0.14 |
| Conduct problems | 0.29 | 0.26 |  | 0.27 | 0.21 |  | 0.24 | 0.17 |
| Substance abuse | 0.06 | 0.14 |  | 0.14 | 0.16 |  | 0.21 | 0.16 |
