# Supplementary material for: The etiological relationship between the general factors of psychopathology and personality; a longitudinal twin study from adolescence into young adulthood
Source: Front Psychol. 2025 Jul 8;16:1564305. doi: 10.3389/fpsyg.2025.1564305 (PMC12279783; doi:10.3389/fpsyg.2025.1564305)
Supplement: Supplementary file 2 [file Table_2.docx]

**Table S2**

*Twin Correlations*

| Variable | 1.  Personality _Wave 1_ | 2.  Personality _Wave 2_ | 3.  Personality _Wave 3_ | 4.  Psychopathology _Wave 1_ | 5.  Psychopathology _Wave 2_ | 6.  Psychopathology _Wave 3_ |
| --- | --- | --- | --- | --- | --- | --- |
|  | MZ correlations | | | | | |
| 1. Personality _Wave 1_ | .57^***^ |  |  |  |  |  |
| 2. Personality _Wave 2_ | .49^***^ | .56^***^ |  |  |  |  |
| 3. Personality _Wave 3_ | .46^***^ | .51^***^ | .53^***^ |  |  |  |
| 4. Psychopathology _Wave 1_ | -.29^***^ | -.25^***^ | -.21^***^ | .61^***^ |  |  |
| 5. Psychopathology _Wave 2_ | -.26^***^ | -.31^***^ | -.27^***^ | .49^***^ | .62^***^ |  |
| 6. Psychopathology _Wave 3_ | -.27^***^ | -.32^***^ | -.35^***^ | .39^***^ | .49^***^ | .55^***^ |
|  | DZ correlations | | | | | |
| 1. Personality _Wave 1_ | .21^***^ |  |  |  |  |  |
| 2. Personality _Wave 2_ | .19^***^ | .23^***^ |  |  |  |  |
| 3. Personality _Wave 3_ | .16^***^ | .19^***^ | .18^***^ |  |  |  |
| 4. Psychopathology _Wave 1_ | -.16^***^ | -.12^***^ | -.11^***^ | .30^***^ |  |  |
| 5. Psychopathology _Wave 2_ | -.13^***^ | -.15^***^ | -.12^***^ | .21^***^ | .27^***^ |  |
| 6. Psychopathology _Wave 3_ | -.11^***^ | -.12^***^ | -.12^***^ | .18^***^ | .23^***^ | .27^***^ |

*Note.* MZ = monozygotic twins; DZ = dizygotic twins.
