# Supplementary material for: The etiological relationship between the general factors of psychopathology and personality; a longitudinal twin study from adolescence into young adulthood
Source: Front Psychol. 2025 Jul 8;16:1564305. doi: 10.3389/fpsyg.2025.1564305 (PMC12279783; doi:10.3389/fpsyg.2025.1564305)
Supplement: Supplementary file 3 [file Table_3.docx]

***Table S3***

*Fit Statistics from the Trivariate Cholesky Decompositions*

| *Model* | | *AIC* | *BIC* |
| --- | --- | --- | --- |
| *Personality* | |  |  |
|  | *ACE* | *16568.7* | *16699.9* |
|  | ***AE*** | ***16556.7*** | ***16655.1*** |
| *Psychopathology* | |  |  |
|  | *ACE* | *18129.4* | *18260.6* |
|  | ***AE*** | ***18118.9*** | ***18217.3*** |

*Note. The best fitting models is indicated in bold. AIC = Akaike’s information criterion; BIC = Bayesian information criterion.*
