# Supplementary material for: The etiological relationship between the general factors of psychopathology and personality; a longitudinal twin study from adolescence into young adulthood
Source: Front Psychol. 2025 Jul 8;16:1564305. doi: 10.3389/fpsyg.2025.1564305 (PMC12279783; doi:10.3389/fpsyg.2025.1564305)
Supplement: Supplementary file 4 [file Table_4.docx]

**Table S4**

*Heritability and Proportion of Non-Shared Environmental Variance Within the Psychopathology and Personality Factors*

|  |  | Non-shared environmental variance (e^2^) |
| --- | --- | --- |
| Variable | Heritability (a^2^) |  |
| Personality _Wave 1_ | .53 [.48, .58] | .47 [.42, .52] |
| Personality _Wave 2_ | .53 [.47, .58] | .47 [.42, .53] |
| Personality _Wave 3_ | .49 [.43, .54] | .51 [.46, .57] |
| Psychopathology _Wave 1_ | .59 [.54, .64] | .41 [.36, .46] |
| Psychopathology _Wave 2_ | .58 [.53, .63] | .42 [.37, .47] |
| Psychopathology _Wave 3_ | .54 [.48, .58] | .46 [.42, .52] |

*Note.* Confidence intervals in square brackets.
